# Emergent management of postpartum hemorrhage for the general and acute care surgeon

**DOI:** 10.1186/1749-7922-4-43

**Published:** 2009-11-25

**Authors:** Allison B Weisbrod, Forest R Sheppard, Mildred R Chernofsky, Charles L Blankenship, Frederick Gage, Gary Wind, Eric A Elster, William A Liston

**Affiliations:** 1 Department of Surgery, National Naval Medical Center, Bethesda, MD, USA; 2 Department of Surgery, Uniformed Services University, Bethesda, MD, USA; 3 Regenerative Medicine, Naval Medical Research Center, Silver Spring, MD, USA; 4 Department of Obstetrics and Gynecology, Walter Reed Army Medical Center, Washington, DC, USA

## Abstract

**Background:**

Postpartum hemorrhage is one of the rare occasions when a general or acute care surgeon may be emergently called to labor and delivery, a situation in which time is limited and the stakes high. Unfortunately, there is generally a paucity of exposure and information available to surgeons regarding this topic: obstetric training is rarely found in contemporary surgical residency curricula and is omitted nearly completely from general and acute care surgery literature and continuing medical education.

**Methods:**

The purpose of this manuscript is to serve as a topic specific review for surgeons and to present a surgeon oriented management algorithm. Medline and Ovid databases were utilized in a comprehensive literature review regarding the management of postpartum hemorrhage and a management algorithm for surgeons developed based upon a collaborative panel of general, acute care, trauma and obstetrical surgeons' review of the literature and expert opinion.

**Results:**

A stepwise approach for surgeons of the medical and surgical interventions utilized to manage and treat postpartum hemorrhage is presented and organized into a basic algorithm.

**Conclusion:**

The manuscript should promote and facilitate a more educated, systematic and effective surgeon response and participation in the management of postpartum hemorrhage.

## Background

Postpartum hemorrhage (PPH) is one of the rare occasions when a general or acute care surgeon may be called to labor and delivery emergently. At the least, this represents entrance into an environment and scenario that for most surgeons is not only foreign but also one in which time is limited and the stakes high. Being prepared to competently participate in the management of severe postpartum hemorrhage necessitates a basic knowledge of pelvic and gynecologic anatomy, the pathophysiology of such hemorrhage and a conceptual algorithm for its management to permit integrated participation with the obstetrical team for efficient and efficacious care of the new mother.

Postpartum hemorrhage may occur in 1-5% of deliveries in developed countries [1,2], and is still the most significant cause of maternal morbidity and mortality [3]. Blood loss following childbirth will vary depending on the type of delivery: vaginal versus cesarean. Classically, PPH has been defined as a blood loss greater than 500 mL after a vaginal delivery and greater than 1000 mL after a cesarean section. These definitions are flawed in that it is recognized that 500 mL is the average blood loss after a vaginal delivery and 1000 mL is the average blood loss after a cesarean [1]. Underestimation of post-delivery blood loss is not uncommon, and is likely contributed to, at least in part, by the ability of healthy pregnant women to lose up one liter of blood acutely without a noticeable drop in hemoglobin or significant hemodynamic change [4,5]. A more useful and accepted definition of PPH is defined as blood loss sufficient to cause hypovolemia, a 10% drop in the hematocrit or requiring transfusion of blood products (regardless of the route of delivery) [5]. PPH of this nature may occur in 4% of vaginal deliveries and up to 6% of cesarean deliveries in developed countries [6-8]. Postpartum hemorrhage may develop in patients with no risk factors; however, reported risk factors include: multiparity, operative deliveries (forceps or vacuum assisted deliveries), previous postpartum hemorrhage, antepartum hemorrhage, prolonged third stage of labor (delivery of the placenta more than 30 minutes after delivery of the fetus), abnormal placentation (placenta previa, accreta or increta), oxytocin use, maternal obesity, and a distended uterus (from a large baby, multifetal gestation or excessive amniotic fluid) [6-9]. In developed countries, the maternal mortality of such hemorrhage has been reported to be on the order of 0.1% of all deliveries [9].

It is the goal of this paper to serve as a refresher and basic fund of knowledge for general surgeons with regard to postpartum hemorrhage so that when called upon to assist in such a scenario, prompt and efficacious assistance may be provided in a spontaneous, educated and systematic manner.

## Call to the General/Acute Care Surgeon

When a significant postpartum hemorrhage occurs, a call may be placed for assistance from a general or acute care surgeon. This call should be considered and responded to as an emergency, synonymous with a cardiopulmonary arrest or trauma alert or activation. There are 3 common clinical scenarios involving acute postpartum hemorrhage (PPH within the first 24 hours from delivery) when a general surgeon or acute care surgeon may be called upon:

1. Most commonly, the patient is in the operating suite in labor and delivery following a cesarean section and a hysterectomy is being considered or performed for PPH that has not responded to the usual medical and surgical measures. These patients likely will be hemodynamically unstable and may be experiencing latent or full-blown disseminated intravascular coagulation (DIC).

2. The second most common scenario will be a patient status post a vaginal delivery who is experiencing PPH refractory to medical measures who has been or is being moved to the labor and delivery operating suite for an operative intervention. Similarly, these patients will be in or near significant hemodynamic compromise and DIC.

3. Lastly, and probably the least likely scenario, is the previous patient, still in the delivery suite. A good number of these patients will respond to medical interventions to control their PPH. This situation is usually handled by obstetrical practitioners, who would try medical measures on their own, or call another obstetrical practitioner.

## Resuscitation

Once significant postpartum hemorrhage has been recognized, resuscitation is performed in parallel to diagnostic efforts. The initial assessment of the patient should be conducted in much the same manner as per Advanced Trauma Life Support (ATLS) guidelines. Certainly, this should be tailored and should take into account what has been and is already underway; however, "ABCs" must be evaluated with interventions provided as needed. If not already in place, appropriate monitors should be attached and monitoring commenced: Continuous pulse oximetry, heart rate and blood pressure measurements (a minimum of every 5 minutes), and a foley catheter, if not in place, should be placed and utilized to monitor urine output. Initial lab studies should be ordered and repeated as needed and at least every 4 hours, to include type & cross for six units of packed red blood cells (PRBCs), chemistry panel, complete blood count (CBC), coagulation panel, and fibrinogen. Unique to the postpartum patient, D-Dimer studies may be sent; however interpretation must take into account that pregnancy itself results in elevated values, therefore limiting its utility [10]. At a minimum two large bore IVs (14 gauge) should be in place and if necessary, central intravenous access and arterial lines should be inserted for central venous pressure monitoring, additional fluid infusion, continuous blood pressure monitoring and ease of subsequent lab draws. Appropriate personnel in the blood bank should be notified early and a massive blood transfusion protocol initiated preemptively if blood transfusions are anticipated.

Fluids should be replaced with the goal of matching all previous losses within the first hour. The rate is then titrated to provide maintenance fluids and make up for continued losses so appropriate vital signs can be maintained. It is prudent to limit fluids to no more than 2 L of crystalloids, 1.5 L of colloid or 2 units of type O-negative blood prior to providing cross-matched blood to the patient [11]. A more accurate assessment of volume loss can be assessed by calculating the patient's blood volume is (8.5-9% of a pregnant woman's body weight) and comparing it to estimated blood loss (determined by changes in pulse, systolic blood pressure and mean arterial pressure) [12]. If bleeding persists with blood loss greater than 40% of estimated patient blood volume, packed red blood cells should be transfused [13]. Early consideration of PRBC transfusion in these patients is warranted due to their baseline moderate hemodilution.

## Examination and Initial Interventions

Establishing a cause of hemorrhage is the first step towards correcting the problem. The most common causes include, in decreasing incidence: uterine atony, retained products of conception, placental abnormalities, uterine inversion, uterine rupture, genital tract trauma and coagulopathies [14]. An initial physical exam is needed to identify atony and to repair lower genital tract trauma, as well as to identify and remove any retained placental tissue.

Uterine atony refers to a floppy, flaccid uterus, one in which the myometrium is unable to contract effectively after the expulsion of the placenta leading to hemorrhage. Bimanual uterine massage should be performed, with one hand in the vagina, and the other hand placed on the abdomen at the level of the uterine fundus to stimulate uterine contraction.

Retained uterine products are the most common cause of delayed (occurring more than 24 hours after birth) post partum hemorrhage [12]. In normal circumstances, uterine contractions expel the placenta within a few minutes of childbirth. The placenta is then examined, to ensure it is intact. Predisposed risk for retained uterine products includes history of previous curettage, cesarean section, multiple births or endometrial infection or injury [15].

If the placenta has not been delivered within 15-30 minutes of childbirth or in cases suspicious for retained placental fragments, the placenta must be retrieved [7]. Golan and colleagues, 1983 [15], showed success of medical management by injecting 10 IU of oxytocin directly into the umbilical vein. Providing bleeding cessation in 10 of 10 patients treated for PPH due to delayed (>30 min) expulsion of placenta. If this umbilical vein injection is bypassed, or not successful, adequate regional anesthesia or general anesthesia should be ensured; current hemostatic parameters should be reassessed with cross-matched blood available, broad spectrum antibiotics administered and an oxytocin drip (40 IU oxytocin in 500 mL of 0.9% saline, at 125 mL/hr) should be started before attempting to remove retained uterine products. The best way to remove retained products is to approach transvaginally, finding the plane between the placenta and uterine wall then gently separate the placental parts from the uterus sweeping the surgeon's fingers in a side-to-side motion. After this has been completed, the uterine cavity should again be checked to ensure it is empty [11].

Injuries to the genital tract may produce severe bleeding, a quantity that may be unexpected to the inexperienced. Optimal repair includes correct positioning of the patient to allow for adequate vision and access of surgical instruments. In order to gain effective control of the bleeding, the injured area should be sutured, starting at the apex of the tear. If the apex cannot be reached, the suture should be started as close to the apex as possible, then, once the remainder of the tear has been approximated, place traction to reach the previously hidden apex. If there is extensive trauma to the vaginal wall, with multiple lacerations, bruising and oozing repairs, vaginal packing to provide hemostasis should be placed and maintained for 12-24 hours [11]. Vaginal packing consists of gauze tape, roller gauze or gauze 4 × 4's that are tied end to end, placed loosely at first, then more tightly in subsequent layers using a ring or dressing forceps to create a mass the size of a softball. It is important to ensure foley catheter has been placed to allow an outlet for urination in addition to monitoring of urine output [16].

## Failure of Hemorrhage Control

If postpartum hemorrhage has not been controlled at this point the patient should be emergently moved to the labor and delivery OR suite, notifying the anesthesia provider, the blood bank (of the possibility for massive transfusion protocol) and the following staff, if available: Staff General/Trauma surgeon, senior general surgery residents, the patient's nurse and any available nurse's assistants. Activation of the hospital's massive transfusion protocol specifies a predetermined ratio of PRBCs, thawed plasma, cryoprecipitate, platelets, and recombinant factor VIIa (rFVIIa) in hopes of preventing the early coagulopathy seen in trauma patients receiving 10 or more units of packed red blood cells [17]. Many hospitals have created their own unique protocol to address this aspect of management, such as Vanderbilt University Medical Center, which has published their hospital's guidelines: for the first round of transfusion, 10 units of non-irradiated, uncrossed packed red blood cells, 4 units of AB negative plasma and 2 units of single donor platelets are sent by the blood bank; then for continued hemorrhage, bundles of blood products are sent containing 6 units of non-irradiated PRBCs, 4 units of thawed plasma and 2 units of single donor platelets [18]. in obstetrical patients if transfusion is needed before type specific or crossmatched blood can be obtained, if possible type-O, Rh-negative blood should be utilized because of future risk of Rh sensitization; however if not readily available Rh-positive blood should not be withheld if clinically required. The surgeon must be aware that hemolytic transfusion reactions with emergency non typed blood can reach up to 5% [19].

## Escalated Medical Management

If initial interventions fail to control postpartum hemorrhage, a stepwise progression of medical therapy is available using uterotonics to facilitate contraction of the uterus.

The first agent used is oxytocin. In the United States, oxytocin is typically administered after delivery of the placenta dosed at 10-20 units in 1000 mL of crystalloid solution, given intravenously (IV) and titrated to an in infusion rate that achieves adequate uterine contractions. Less commonly, it can be given intramuscularly (IM) or intrauterine (IU). It is common practice to double the oxytocin in PPH, i.e., 40 units in 1 L, and safety/efficacy has been documented up to 80 units per liter of crystalloid [20]. Oxytocin is not bolused, as boluses can cause hypotension. Excessive oxytocin can cause water intoxication, as it resembles antidiuretic hormone.

If there is not adequate uterine tone with oxytocin, the second line agent used will depend on the medications' side effects and contraindications. Two classes of drugs are available: ergot alkaloids (methylergonovine) or prostaglandins (PGF2α, PGE1, and PGE2). Methylergonovine may be used, dosed as 0.2 mg IM and repeated 2-4 hrs later, as long as the patient does not have hypertension or preeclampsia. If the patient has contraindications to methylergonovine or if the hemorrhage is still non-responsive, 250 μg of 15-methylprostagandin F2α may be injected intramuscularly (IM) up to 3 times at 15-20 minute intervals (maximum dose 2 mg) [21]. Appropriate injection points include thigh, gluteal muscle or directly into the myometrium. While these drugs are commonly administered without incident, the patient should be monitored for a reflexive response of hypotension and circulatory collapse [22]. Response usually occurs within minutes with clinical trials showing a 75% response rate to a single dose of 15-methylprostaglandin F2α increasing to a 95% response after three doses [12]. PGF2α is contraindicated in asthma and hypertension patients, as it can cause significant broncho-constriction and elevated blood pressures. It's side effect profile includes diarrhea, nausea, vomiting and fever. More recently, PGE1 (misoprostol) has shown promise and is being used more frequently, due to its lack of contraindications and minimal side effects of tachycardia and fever. (A single dose of 1000 μg may be administered rectally [23]. A final option is PGE2, which is administered 20 mg rectally with repetition, as necessary every 2 hours. Unfortunately, it has an unfavorable side effect profile that includes fever, chills, nausea, vomiting, diarrhea and headaches [24].

Although not commonly described in discussions of post-partum hemorrhage management, Lurie and colleagues, 1997 [25], described the cessation of uterine bleeding after injecting 1 mL (5IU) of vasopressin in 19 mL of normal saline subendometrially. Throughout these treatments, staff should continue to administer bimanual uterine compressions [11]. If all of the medical treatments have failed and all other causes of post-partum hemorrhage have been excluded, treatment should progress to surgical options.

## Uterine Tamponade

Pressure and tamponade are commonly used methods to control bleeding. Uterine packing applies these principles, making it a popular technique for over a century, whereas balloon tamponade is a more recent development.

Uterine packing is a quick, viable option to create hemostasis. Critics' concerns address the large quantities of blood that may be absorbed by the pack or hidden behind the pack before hospital staff can recognize that bleeding has continued. It may be performed in one of two acceptable transvaginal methods; both using non-medicated, dry gauze. The first technique of uterine packing employs a tubular packer, such as the Holmes or Torpin packer. The cervix is exposed, then grasped securely with a sponge forceps or a tenaculum. The stylet or plunger of the packer is used to insert the gauze into the uterus until it is packed tightly all the way to the introitus. In the second technique, a packing or dressing forceps is used to introduce the gauze into the uterus, using short strokes and taking care not to remove the tips of the forceps until the uterus and vagina are tightly packed. Broad-spectrum antibiotics should always be used prophylactically to prevent complications from sepsis. The pack can be left in place and managed in the same fashion as intraabdominal packing for abdominal damage control. To remove the pack, the patient should first receive an anxiolytic, such as 10 mg of IV diazepam before slowly pulling the gauze out. Oxytocin, administered for 12-24 hours after the pack is removed, provides adequate muscle contraction, thus preventing the re-initiation of bleeding [26].

An alternate method is balloon tamponade. Balloon tamponade has been used in different scenarios of uncontrolled bleeding, including esophageal varices, massive bladder hemorrhage and bleeding associated with prostatectomy. The general idea is to insert a sterilized balloon into the uterine cavity, then fill the balloon with warm water to see if additional pressure can control the patient's hemorrhage. Four methods have been described in the literature.

In the original description of the 'tamponade test', a Sengstaken-Blakemore tube is used, prepared by cutting off the portion of the tube distal to the stomach balloon. Two pair of sponge forceps are needed: the first, used to grasp the anterior lip of the cervix and facilitates the placement of the balloon into the uterine cavity, held by the second pair of forceps. Warm saline was used to fill the balloon until it was visible at the cervical canal - using approximately 50-300 mL of fluid [27-29].

Johanson, et al, 2001 [30], described the same process using a Rusch balloon catheter, a type of urologic hydrostatic balloon catheter. The patient is placed in the Lloyd Davies position and a weighted speculum is used to insert the balloon into the uterine cavity. The balloon is inflated through the drainage port, using approximately 400-500 mL of warm saline.

Bakri, et al., 2001 [31], developed 'the tamponade balloon' specifically for lower-uterine post-partum hemorrhage. The patient is placed in the lithotomy, or 'frog-leg' position and the distal end of the balloon catheter is inserted into the uterus through the cervix. A speculum is used to place vaginal packing, then the balloon is inflated with 250-500 mL of warm water. A Foley catheter may be used for this maneuver, using the largest caliber Foley catheter after first removing the portion of the catheter beyond the balloon attachment. The catheter is introduced through the cervix to the uterus, and the balloon is filled with adequate fluid to provide a tamponade effect - 5 to 40 mL has been described as an appropriate amount. Clamping the catheter will provide additional pressure.

A successful tamponade demonstrates decreased or minimal bleeding after balloon inflation, thus terminating the need for surgical treatment. To help maintain the placement of the balloon, the upper vagina is packed with roller gauze. The previously placed Foley catheter should be kept in place to facilitate bladder drainage. Additionally, the previously started oxytocin infusion should be maintained for 12-24 hours and broad spectrum antibiotics are continued for three days to decrease the patient's risk for sepsis. After 24 hours of monitoring without subsequent bleeding, hemostatic interventions are removed in a step-wise manner. First the balloon is deflated but left in place. If no bleeding is seen after 30 minutes of observation, the oxytocin infusion is stopped and the patient is again monitored for 30 minutes. The balloon and vaginal packing may be removed if all bleeding has ceased [11].

## Operative Management

For surgical management, the patient is generally placed under general anesthesia in the Lloyd Davies position (lithotomy position with trendelenberg) [11]; although Pal and colleagues, 2003 [32], describe success with supine positioning. If child birth occurred via caesarean section, and there is ongoing bleeding, one can directly carry out the surgical maneuvers described below through the open incision. If PPH occurs in the recovery room after a completed cesarean section, the patient should be emergently returned to the OR, and the skin incision is re-opened. If PPH occurs following a vaginal delivery a Pfannenstiel or midline incision is utilized to rapidly access the uterus through the abdomen [11]. Once access is attained, multiple surgical options are available, to include undersuturing venous sinuses, a variety of compression suture techniques and selective arterial ligation.

### Undersuturing

One of the simplest surgical solutions to stop post-partum hemorrhage is the undersuture. The thinness of the tissue in the lower uterine segment and the narrowed section of the cervical canal often causes difficulty, due to the friability of the area. Because of this, full-thickness sutures work best. Horizontal sutures are placed across and below the bleeding points. It is important not to obliterate the OS or the cervical canal to allow residual blood to drain through the vagina [11].

### Compression Sutures

Compression sutures are a recent innovation used to address post-partum hemorrhage. The original technique was the B-Lynch suture, created by Dr. B-Lynch, a British Obstetrician/Gynecologist [33]. Adaptations of this technique include the square suture and the modified B-Lynch sutures, created by Drs. Cho (2000) [34] and Hayman (2002) [35], respectively. Since these are recent techniques, published evidence is mostly limited to case reports and series. In his 2007 article, Baskett offers results of a 7-year study of compression sutures, all done at the time of cesarean delivery, showing that compression sutures were able to control bleeding in 23 of 28 (82%) of women, thereby preventing hysterectomy. Of these women, seven were able to have subsequent uncomplicated term pregnancies [36].

### B-Lynch Suture

**Figure 1:**
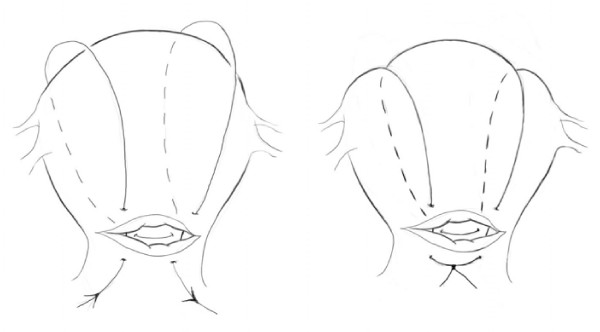
**B-Lynch Suture Technique: The B-Lynch Suture Technique was the originally described compression suture **[27], **providing a simple and fertility-sparing option for treatment of post-partum hemorrhage**.

The B-Lynch suture technique was introduced in 1997 as a type of vertical brace suture used for diffuse uterine bleeding. It works by opposing the anterior and posterior walls of the uterus [33]. The utility of the B-Lynch suture is attributed to its simplicity, safety, ability to preserve life, the uterus and fertility with the benefit of immediate evaluation of hemostatic success [37] Of the 60 published case reports in which the B-Lynch suture was used, only one negative outcome (uterine necrosis) was documented [38]. Details regarding this stitch are as follows, and can be seen at Dr. B-Lynch's website: http://www.cblynch.com/video.html. 33 the stitch is depicted in Figure 1. Prior to utilization of this technique the uterus should be externalized and bimanual compression applied to determine the value of the B-Lynch suture. If hemostasis is achieved with such compression, the surgeon should proceed with this technique.

A No. 2 chromic catgut suture is used to enter the uterus 3 cm from the right lateral border and 3 cm below the right lower edge of the uterine incision. The suture is passed through the uterine cavity, exiting 3 cm above and 4 cm medial to the lateral border at the upper margin of the uterus. The suture is run externally over the anterior, fundal, and then posterior surfaces of the uterus in a plane 3-4 cm medial to the right cornual border before the needle is reinserted at a point in the posterior wall that corresponds to the anterior uterine incision. A surgical assistant may apply bimanual uterine compression to aid in pulling the suture under moderate tension.

Once the right side of the uterus has been compressed by the first half of the B-Lynch suture, the needle is passed laterally to the left side of the cavity, exiting the posterior wall of the uterus in a horizontal plane to the posterior wall entry point. The suture is threaded over the posterior, fundal and anterior surfaces in a plane 3-4 cm medial to the left cornual border before re-entering the uterine cavity anteriorly at a point 3 cm above the uterine incision and 4 cm from the lateral border; effectively completing the first half of the stitch in the opposite direction. Again, it is useful to have an assistant present to apply bimanual uterine compression while the stitch is pulled under moderate tension. The suture is passed inferior to the uterine incision, and then emerges through the anterior uterine wall at a point 3 cm below the uterine incision and 3 cm medial to the lateral border of the uterine wall. The stitch is completed by tying the right and left sides of the suture on the anterior surface of the uterus inferior to the uterine incision. The uterine incision, followed by the abdominal wall is then closed similar to the closure of a cesarean section.

### Square Suture

**Figure 2:**
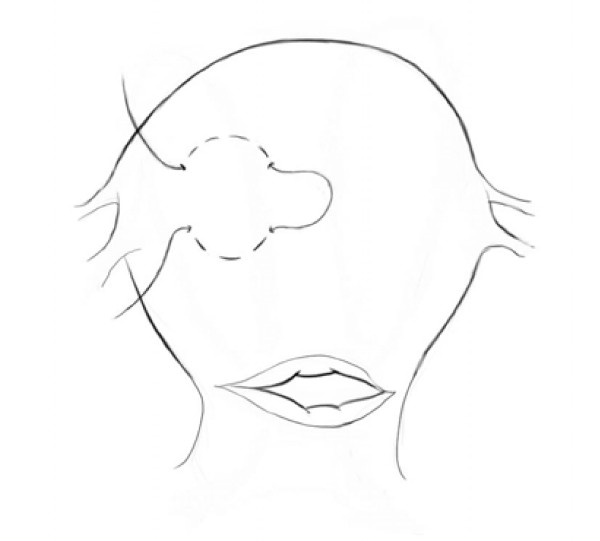
**Square Suture Technique: The Square Suture technique was created and described by Cho and colleagues **[28], **offering an alternative to the B-Lynch technique**. This suture is considered to be a safer option as the uterine vessels do not cross the anatomy where the stitch is placed.

Cho and colleagues, 2000 [34], described another suturing technique used to control bleeding due to post-partum hemorrhage - the square suture (See Figure 2). This simple stitch offers additional safety to less experienced surgeons since the ureters and great vessels are not at risk [38]. To perform the square suture technique, a straight needle with a No. 1 chromic catgut stitch is threaded through both the anterior and posterior uterine walls at an area of heavy bleeding. The return entry point can be chosen at any site 2-3 cm from where the suture was initially passed. An additional stitch is placed through both walls - first anterior to posterior, then posterior to anterior to complete the shape of a square, each side measuring 2-3 cm. The suture is completed with a tightly tied knot. If bleeding is attributed to uterine atony, a total of 4-5 square sutures should be placed [34]. In the case of placenta accreta or previa, (types of abnormal placentation where the placenta lacks a clear plane to separate from the uterus, previa: no plane between the placenta and the myometrium, accreta: placenta has partially invaded the myometrium), 2-3 square sutures should be placed in the areas of heaviest bleeding [11].

### Modified B-Lynch Suture

**Figure 3:**
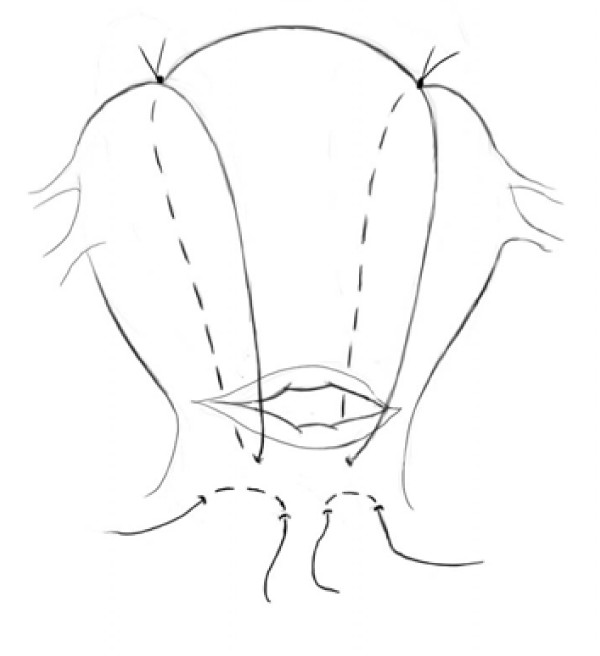
**Modified B-Lynch Suture: The Modified B-Lynch Suture **[29]**is an adaptation of the B-Lynch suture, used for cases in which the source of bleeding is identified to be contained primarily within the fundus of the uterus**.

Hayman, et al., 2002 [35], described a modified version of the B-Lynch suture after a case of placenta previa accreta. In the case for which he adapted the stitch, bimanual compression only controlled fundal bleeding, not cervical hemorrhage. The cervical portion of the uterus needed direct external anterior to posterior compression to control bleeding. This lead to the development of the isthmic-cervical apposition suture in addition to the modified B-Lynch suture [39]. (See Figure 3) Advantages include added simplicity and avoidance of uterine incision [38].

To perform this stitch, a straight needle with a 2-Dexon suture is inserted into the uterus above the bladder reflection 2 cm medial to the lateral border of the lower uterine segment and 3 cm below the left lower edge of the uterine incision. The needle is then threaded through to the posterior wall of uterus, then returned from posterior to anterior wall at a point 1-2 cm medial to the first pass of the suture and both ends were tied on the anterior aspect of the anterior wall. The stitch is then repeated on the same horizontal plane on the right side of the lower uterine segment [35].

To control bleeding in the body of the fundus, the modified brace suture is added. A No. 2 chromic cat gut suture is placed in the anterior wall of the uterus and passed through the posterior wall of the uterus, just superior to the isthmic-cervical apposition suture. The ends of the suture are tied using a three-knot technique at the fundus, 3-4 cm medial to the cornua while external compression is performed by an assistant. An identical stitch is performed on the contralateral side. If this doesn't control the bleeding, horizontal compression sutures may be added to the modified B-Lynch sutures [35].

Throughout this technique, it is important to keep the lower cervical segment and the OS patent in order to allow drainage of blood from the cavity. Afterwards, the bladders, ureters and bowel must be inspected to exclude trauma [35].

### Uterine Artery Ligation

Uterine artery ligation is one of the easiest and most effective surgical measures to control PPH. It is relatively safe, can be performed easily, and allows for future childbearing. The uterine arteries supply 90% of the blood to the uterus; therefore, ligation drastically decreases blood flow and subsequent blood loss [11]. Despite this percentage, the surgeon should not worry about resultant uterine necrosis, as adequate blood supply is still available [22].

This procedure is performed as follows. First the vesicouterine fold of peritoneum is identified and incised transversely in order to mobilize the bladder inferiorly. Next, the uterus is externalized for full exposure in order to identify an avascular window in the broad ligament. If an avascular area is not readily apparent, the surgeon may use the lateral border of the uterus. A No. 1 chromic catgut or polyglycolic suture should be used to make a posterior to anterior stitch through the myometrium at a site 2-3 cm medial to the uterine artery. The needle is returned anterior to posterior through the avascular window at a site just below the level of the utero-vesical peritoneal reflection. The two ends are tied securely, completing the ligation. The ureters, bladder and bowel should all be inspected for inadvertent trauma before repeating the procedure on the contralateral uterine artery [11].

### Utero-Ovarian Artery Anastomosis Ligation

**Figure 4:**
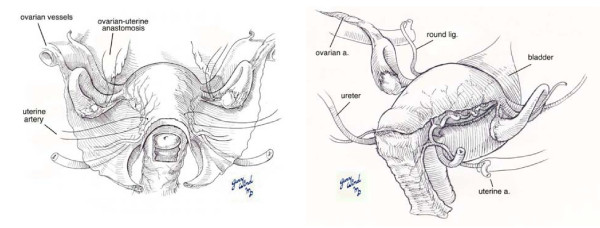
**Significant Uterine Vessels**. The uterine artery, the anastomosis of the utero-ovarian artery and the hypogastric artery are all acceptable places to perform an arterial ligation.

Ligation of the utero-ovarian artery anastomosis is similar to the uterine artery ligation. An avascular area is identified in the meso-ovarium, just inferior to the utero-ovarian ligament. Using this site as a securing point, a ligature is placed around the utero-ovarian anastomosis. The ovaries should be checked to ensure ovarian blood supply has not been compromised [11]. Please refer to Figure 4 for an anatomic depiction.

### Internal Iliac Artery (Hypogastric Artery) Ligation

Internal Iliac artery ligation is the next step in treatment. Bilateral ligation of the vaginal branch decreases pulse pressure in the distal arteries by 85%, improving. Unfortunately this procedure has a low success rate, estimated at 40%, mostly attributed to the late stage at which the ligation is attempted and that it is frequently complicated by hematoma formation and tissue edema that obscure the anatomy [11].

The steps to perform the internal iliac artery ligation are as follows. An 8-10 cm incision is made in the peritoneum parallel and lateral to the ureter which opens the retroperitoneal space. The peritoneal flap with the ureter is retracted medially so that the internal iliac artery may be dissected out and the posterior branch can be identified. Next, the posterior branch of the internal iliac artery is separated from the internal iliac vein and a right-angled clamp is used to place two ligatures around each of the vessels. It is important to check the external iliac artery to confirm that adequate pulse pressure is present for perfusion of distal branches. It is also important to inspect the ureters for signs of trauma. Once these are completed, the steps are repeated on the contralateral side [11]. Please refer to Figure 4 for an anatomic depiction. Complications of this procedure can be severe, including ischemic damage to the pelvis, decreased blood flow to the gluteal muscles (if the ligation is performed above the branch point of the posterior branch, or injury to the iliac vessels [11].

### Hysterectomy

Hysterectomy is the last line of treatment available for treating post-partum hemorrhage attributed to uterine bleeding. It is only used for hemorrhage unresponsive to other management attempts, as it removes the patient's option to bear additional children [40]. Recently, the subtotal hysterectomy has become a preferable procedure in this situation. It is quicker, associated with less blood loss, reduced intra- & postoperative complications and reduced need for further blood transfusion [41]. However, if the bleeding source is found in the lower segment of the uterus, a total hysterectomy is needed [11]. Unfortunately, both subtotal and total hysterectomy completed for post-partum hemorrhage is associated with high rates of maternal mortality [40].

A midline or transverse incision is used to open the abdomen. The bowels are packed out of the operating field to protect them from injury. The round ligaments are identified bilaterally, then clamped, divided and ligated. Next, the posterior leaf of the broad ligament is identified. It is perforated just inferior to the Fallopian tubes so that the utero-ovarian ligament and ovarian vessel can be clamped, divided and ligated. This step is repeated on the opposite side. Now, the broad ligament is detached: the posterior leaf is divided up to the uterosacral ligaments, and the anterior leaf is divided down to the superior margin of the bladder. The bladder is mobilized by making an incision in the vesicouterine fold of the peritoneum then bluntly dissecting the fascia away. By dissecting with a downward placement of tissue, the ureters should be pushed out of the operating field and out of harm's way. Next, the uterine vessels are identified bilaterally. Each is clamped close to the uterus so they may be divided and ligated. If a subtotal hysterectomy is adequate, the procedure is completed by transecting the cervix and closing the residual stump with interrupted stitches. If a total hysterectomy is necessary, the bladder is dissected away from the cervix until the superior portion of the vagina can be identified. The cardinal ligaments are located, again clamping each before their division and ligation. Finally, the cervix is detached from the vagina by cutting as close to the cervical margin as possible. The cardinal ligaments are used to secure the lateral vaginal fornices prior to suturing the vaginal vault closed [11].

## Selective Arterial Embolization

Selective arterial embolization has been well documented throughout the literature as a means of controlling post-partum hemorrhage. It is recommended as an alternative to surgical therapy with success rates of 85-100%. The uterine artery is the most commonly embolized vessel, followed by the pudendal, hypogastric, obturator, vaginal and cervical arteries [42]. Unfortunately, the utility of selective arterial embolization is often limited to a small number of hospitals where a trained, available interventional radiologist is present [11].

Local anesthesia or an epidural should be administered prior to the initiation of embolization by cannulization of the femoral artery [14]. The catheter is advanced under fluoroscopy proximal to the point of bleeding and an angiogram is done to confirm the bleeding source. The bleeding vessel(s) is/are catheterized to control the hemorrhage [43]. During the embolization, an absorbable gel sponge, usually reabsorbed within 10 days, may be used [44]. Vessels should always be embolized bilaterally, as a unilateral embolization can increase the risk of further bleeding by secondary recanalization of collateral branches [14].

## Bleeding Stops

The surgeon may change to a consultant role once control of the bleeding has occurred and the patient has been stabilized. The patient should be admitted to an intensive care unit for close monitoring until stability has been assured.

## Conclusion

**Figure 5:**
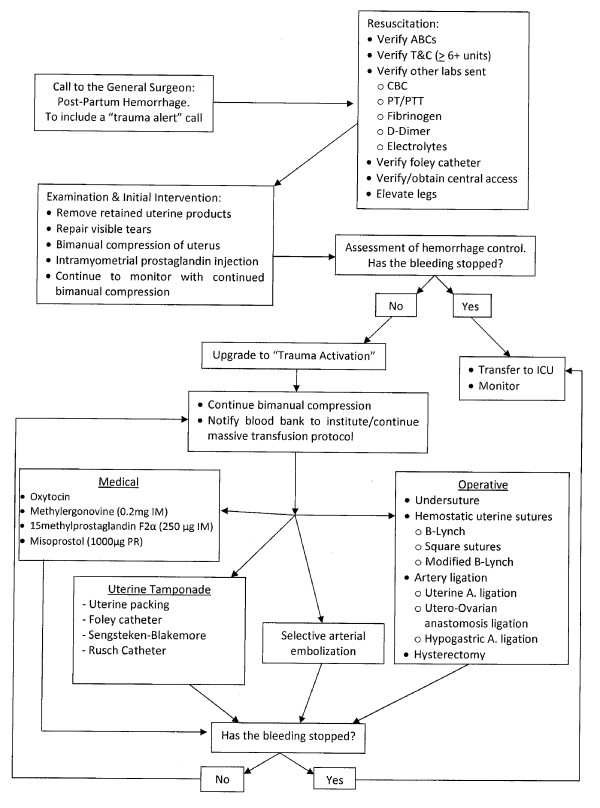
**Algorithm for Management of Post-Partum Hemorrhage**. This figure provides a step-wise chart depicting timely choices for the management of post-partum hemorrhage.

General and acute care surgeons likely will be called emergently to labor and delivery to render assistance for PPH at some point in their careers. The point, at which a surgeon is called, can be anticipated to be later rather than earlier, at a point where operative intervention is being initialized or already underway. Most likely the medical management and non-operative measures presented will not be administered by a surgeon; however, practice and event dynamics will ultimately determine the situation encountered and therefore the knowledge of this information prudent. Though the specific management of severe postpartum hemorrhage is seldom addressed in surgical education and literature, the application of commonly practiced surgical strategies in combination with a basic knowledge PPH specific etiologies, physiology and interventions permits surgeons to efficiently and efficaciously participate in the care of these patients. For our colleagues to have a quick reference guide a flow sheet is available in Figure 5.

## Competing interests

The authors declare that they have no competing interests.

## Authors' contributions

AW collected data, drafted the manuscript and developed the illustrations and figures. FS conceived the initial idea and design of the study, and drafted the manuscript. MC reviewed and assisted with the critical revisions. CB conceived the initial idea and design of the study, reviewed and assisted with the critical revisions. FG assisted with data collection and final edits to manuscript. GW reviewed and assisted with the critical revisions. EE reviewed and assisted with the critical revisions. WL conceived the initial idea, reviewed and assisted with the critical revisions and oversaw project to completion. All authors have read and approved the final manuscript.
